# Controlled automated reperfusion of the whole body after 120 minutes of Cardiopulmonary resuscitation: first clinical report

**DOI:** 10.1186/s13049-017-0412-y

**Published:** 2017-07-10

**Authors:** Georg Trummer, Alexander Supady, Friedhelm Beyersdorf, Christian Scherer, Tobias Wengenmayer, Markus Umhau, Christoph Benk

**Affiliations:** 1 Cardiovascular Surgery, Heart Center Freiburg University, Hugstetter Str. 55, 79106 Freiburg, Germany; 2 Cardiology and Angiology I, Heart Center Freiburg University, Hugstetter Str. 55, 79106 Freiburg, Germany; 3 Institute for Cell and Gene Therapy, University Hospital Freiburg, Hugstetter Str. 55, 79104 Freiburg, Germany; 4 Cardiovascular Surgery, Department of Cardiovascular Surgery, Heart Center Freiburg University, Hugstetter Str. 55, D-79104 Freiburg, Germany

**Keywords:** Cardiopulmonary resuscitation, ECPR, Controlled reperfusion, Reperfusion injury, Extracorporeal circulation, ACLS, ECLS, VA-ECMO, Spinal ischemia

## Abstract

**Background:**

Cardiopulmonary resuscitation (CPR) is associated with a high mortality rate. Furthermore, the few survivors often have severe, persistent cerebral dysfunction. A potential cause for this unsatisfactory outcome after CPR is the combination of cardiac arrest (ischemia) and the inability to restore adequate hemodynamics during conventional CPR (reperfusion), resulting in ischemia/reperfusion injury of the whole body. Therefore we developed a concept counteracting this ischemia/reperfusion injury during the process of CPR.

**Case presentation:**

We present data from a patient, in whom the concept of a novel controlled automated reperfusion of the whole body (CARL) was applied after 120 min of CPR under normothermic conditions. The patient survived without cerebral deficits and showed full recovery of all organs after prolonged cardiac arrest (CA) except for the spinal cord, where a defect at the level of the 11th thoracic vertebra caused partial loss of motoric function of the legs.

**Conclusion:**

This is the first reported clinical application of CARL after CA. The implementation of CARL resulted in unexpected survival and recovery after prolonged normothermic CA and CPR. In synopsis with the preclinical experience in pigs this case shows, that the new concept of CARL treating ischemia/reperfusion during the CPR may be an important element within the future treatment of CA.

**Trial Registration:**

Trial was retrospectively registered in the “German Clinical Trials Register” (www.germanctr.de) under No.: DRKS00005773 on July 28th, 2015.

## Background

Despite continuous efforts, cardiopulmonary resuscitation (CPR) is still associated with high mortality and many survivors suffer from neurological complication [1–3]. Currently, re-establishment of circulation is the main goal in patients undergoing CPR, either manually or with the help of extracorporeal circulatory support devices [4–8]. However, from a pathophysiological point of view, an individual who experiences acute circulatory arrest (CA) requires individualized, continuous reperfusion in order to limit generalized ischemia/reperfusion injury to the whole body [9, 10]. All currently available CPR methods only provide some sort of hemodynamic support, leaving a substantial gap in the therapeutic approach of the life-threatening situation of acute CA. On the basis of this important clinical demand, we developed in preclinical studies a novel whole-body reperfusion protocol (Controlled Automated Reperfusion of the whoLe body—CARL) over the last 10 years [11–13]. After approval of the ethics-committee, CARL has been used in a total of 13 patients with extremely prolonged CPR (unpublished data). The patient discussed in the current case report was the 4th in the row of these 13 patients, distinguished from the other cases by the fact that it was a) the first surviving patient with cerebral recovery treated with CARL, b) the patient with the longest CPR-duration (120 min) and c) the patient with the lowest arterial blood-pH (6.8) with survival and cerebral recovery thereafter. The following report describes the course of this a patient undergoing extremely prolonged CPR with subsequent CARL-therapy.

## Case presentation

We report the case of a 44-year-old woman who underwent CPR for 120 min after CA followed by CARL for 60 min. The patient experienced an acute onset of severe chest pain at her home that was followed by CA. Family members called emergency services and started basic life-support procedures. When the medical team arrived, the patient was not responsive to several cycles of advanced circulatory life support. In this devastating situation, the onsite team decided to transport the patient to our medical center to obtain access to a mechanical circulatory support device. During transport, the medical team continued CPR with chest compressions using the LUCAS™-system (Physio-Control; Lund/Sweden).

The patient arrived at the hospital 90 min after the initial CA. Under the conditions of ongoing CPR, her arterial blood pH at this time was 6.8. Decision for CARL was made by an interdisciplinary team of a cardiologist, cardiovascular surgeon and an intensive-care physician and cannulation of the femoral artery and vein in preparation for the CARL treatment was started immediately. However, access to the very small and spastic femoral artery was difficult and time-consuming. Finally, after successful femoral cannulation (17 Ch arterial, 23 Ch venous; HLS cannula Maquet, Rastatt/Germany) and a total time of exactly 120 min of CPR, blood circulation was provided via a new type of extracorporeal circulation device (controlled, integrated resuscitation device [CIRD]). This device was developed by our group in order to facilitate CARL, i.e. treatment of the previously ischemic whole body. Chest compressions were terminated with the start of the CIRD. In order to prevent ischemia of the leg, an additional sheath (8.5 Ch., Arrow, Everett/USA) was been inserted into the femoral artery distal of the arterial cannula and connected to the arterial cannula.

**Fig. 1:**
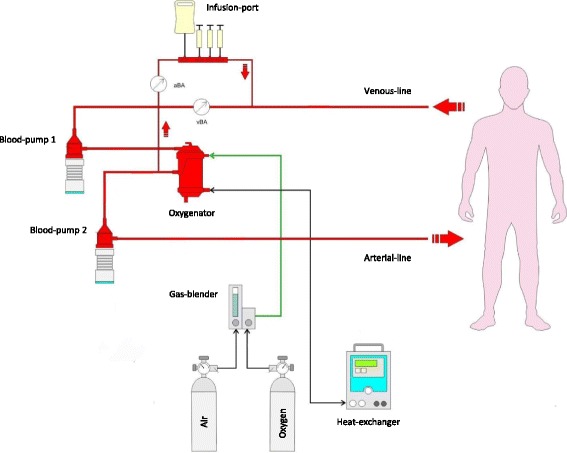
Schematic outline of the Controlled Integrated Resuscitation Device (CIRD) for the application of CARL. The Controlled Integrated Resuscitation Device (CIRD 1.0; ResuSciTec GmbH, Freiburg/Germany) provides continuous venous and arterial blood - gas monitoring (vBA and aBA). Gas-mixture and flow are adapted accordingly in a near closed-loop- fashion. Two blood pumps are used to provide high and pulsatile blood-flow during the reperfusion process while hypothermia is applied via the oxygenator offering immediate temperature-control of the body. The infusion-port on the venous-side of the CIRD is designed for fluid-replacement and supportive medication during the reperfusion-process

The CIRD (CIRD 1.0, ResuSciTec GmbH, Freiburg/Germany) was used to apply the novel post-cardiac arrest reperfusion protocol CARL, which has been designed to limit the extent of ischemia/reperfusion injury of the whole body (Fig. 1). The principle of CARL is to modify the initial conditions of reperfusion (e.g. high arterial blood pressure, pulsatile blood flow, immediate mild hypothermia) and adapt the composition of the initial blood (reperfusate) (e.g. pH-stat, limited arterial oxygen contents, hyperosmolarity, hypocalcemia) to allow the damaged organs to recover. The CIRD circuit was primed with a hyperosmolar priming solution (including albumin, mannitol, sodium-citrate and lidocaine) to provide maximum organ protection after CA.

After initiation of the CIRD, the ongoing and previously uncorrectable ventricular fibrillation was converted into asystole with the application of a bolus of intravenous potassium analogue secondary cardioplegia used in cardiac surgery. Shortly thereafter, the heart regained a stable sinus rhythm. Immediately after initiation of CARL-therapy, coronary angiography was done via the contralateral femoral artery. Parallel to the CARL therapy, an acute occlusion of the proximal left anterior descending coronary artery was diagnosed. PCI was performed and a drug-eluting coronary stent was implanted. CARL was applied for 60 min, followed by continuous extracorporeal circulatory support by the CIRD for 3 days.

**Fig. 2:**
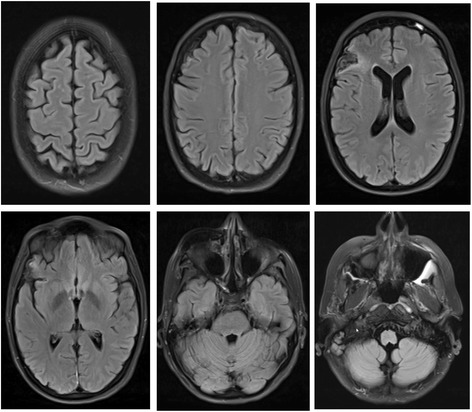
T2-weighted MR of the Head 17 Days after Cardiopulmonary Resuscitation. Selected t2-weighted axial slices of the head 17 days after cardiopulmonary resuscitation. Except for a, most likely preexisting, small cortical defect of the right frontal lobe (upper row, right image) the MRI of the brain shows normal findings

In the ensuing days, organ function recovered stepwise. The reestablishment of satisfactory pulmonary and myocardial function allowed removal of the CIRD 3 days after CPR. The need for mechanical ventilation was prolonged due to concomitant pneumonia most likely related to aspiration during CPR. However, after 12 days, the patient was separated from the ventilator. She regained full consciousness and showed no signs of cerebral deficits. These findings were consistent with the findings in the MRI scans of the brain (Fig. 2). Beyond that, the patient showed signs of spinal injury with a consecutive loss of motor function of the legs with preserved sensory function. MRI-scans of the spinal cord revealed a defect at the level of the 11th thoracic vertebra, most likely related to insufficient perfusion of the spinal vessels during the prolonged period of CA and the subsequent CPR for 120 min. The patient was discharged 21 days after CPR to a neurological rehabilitation facility. One year later the patient experiences a good quality of life without manifest deficits of cerebral function and with recurring motor function of the legs and the ability to walk again.

## Discussion

Full recovery of cerebral function after 120 min of continuous CPR following CA in a normothermic setting is an extremely rare event [14]. The course of this case indicates the potential of CARL. However, the process of CPR, somewhat typical in these cases, deserves closer attention. First of all, the question arises of how long resuscitative attempts should be extended before they are declared unsuccessful. The relevant CPR guidelines are limited regarding CPR duration and time-frames, despite the common practice in an out-of-hospital setting to perform CPR for 30 to 45 min before considering termination in non-responsive patients [15, 16]. Current CPR guidelines focus on optimized perfusion-flow and pressure generated by accurately performed chest compressions [6, 17]. Medical devices (e.g. LUCAS™, Physio-Control; Lund, Sweden) have been developed to substitute high-tech performance for human efforts and support transportation. Although these devices provide a continuously improving quality of manual chest compressions, possible risks of injuring lung, heart, liver, spleen and chest wall have to be considered [18, 19]. Even though the patient was transported using a compression device, the severe metabolic acidosis with high lactate levels and low pH at the time of arrival were indicative of severe ischemia followed by a continued, generalized low-flow/low-pressure situation..

The profound ischemia associated with CA is generally followed by severe cellular injury at the beginning of reperfusion. This triad of symptoms is known as “post-resuscitation syndrome” and has been a major challenge in the treatment of these patients [20, 21]. The therapeutic approach of CARL was developed to limit these detrimental effects. CARL is based mainly on continuous adjustments of reperfusion conditions with the recirculating blood according to the individual readings of the patient. An exemplary and immediate effect of this strategy is the generation of a comparably high and pulsatile blood pressure during extracorporeal circulation. Despite the initial, profound ischemia, which is frequently followed by severe vasoplegia, only low-dose norepinephrine was necessary in this case during the first 60 min of CARL therapy. Supported by the current guidelines recommending extracorporeal circulatory support in CPR, a detailed description of the CARL-protocol became available as part of a standard operating procedure (SOP) within the Heart Center of the Freiburg University before its first use in patients [17, 20] The spinal cord injury underlines the severity of the ischemia caused by the CA and the 120 min of CPR, which obviously resulted in this neurological complication. Spinal cord ischemia after CA has been described previously [22, 23] and is a sign of severe ischemia that is obviously neglected after CPR. The superb clinical outcome of this patient with no neurological deficits after a follow-up of 1 year shows the potential for this novel treatment after CA.

### Limitations

We are aware of the fact, that the result of this case only demonstrates one narrow example and may be not extrapolated generally to other patients. However, the above mentioned reasons are supporting the exceptional character of the medical course. Beyond that, the case report has been performed and evaluated with attention to accepted methodical elements for case studies like significance of the case, unusual general public interest, well-balanced discussion based on the relevant literature and different perspectives regarding the treatment of the medical entity [24].

## Conclusion

In conclusion, this is the first reported clinical application of CARL after CA. The patient suffered a severe ischemic insult after 120 min of conventional CPR following CA: She had a pH of 6.8 and a temporary spinal cord injury. The implementation of CARL using the CIRD −1.0 - system resulted in unexpected survival along with recovery from a spinal cord injury after prolonged CA and CPR. The clinical application of CARL is based on our preclinical research over many years [11–13]. In the near future, we will further improve our CARL technique and develop a mobile CIRD system (CIRD 2.0) that will allow us to start CARL treatment even outside the hospital.
